# The Effect of Viniferin on Liver Cancer: Research Based on Network Pharmacology, Molecular Docking and Molecular Dynamics Simulation

**DOI:** 10.3390/medsci14010130

**Published:** 2026-03-11

**Authors:** Saowanee Maungchanburi, Onwara Wongmek, Poolsak Baitahay, Asron Saweak, Maroof Wangkaranae, Wanmai Kongwattananon, Suphasarang Sirirattanakul, Moragot Chatatikun, Atthaphong Phongphithakchai, Jason C. Huang, Aman Tedasen, Chutima Jansakun

**Affiliations:** 1 Department of Biomedical Sciences and Biomedical Engineering, Faculty of Medicine, Prince of Songkla University, Hat Yai 90110, Songkhla, Thailand; msaowane@medicine.psu.ac.th; 2 School of Allied Health Sciences, Walailak University, Tha Sala 80161, Nakhon Si Thammarat, Thailand; onwara.wo@mail.wu.ac.th (O.W.); poolsak.ba@mail.wu.ac.th (P.B.); asron.sa@mail.wu.ac.th (A.S.); maroof.wa@mail.wu.ac.th (M.W.); wanmai.ko@mail.wu.ac.th (W.K.); suphasarang.si@wu.ac.th (S.S.); moragot.ch@wu.ac.th (M.C.); aman.te@wu.ac.th (A.T.); 3 Research Excellence Center for Innovation and Health Products, Walailak University, Tha Sala 80161, Nakhon Si Thammarat, Thailand; 4 Nephrology Unit, Division of Internal Medicine, Faculty of Medicine, Prince of Songkla University, Hat Yai 90110, Songkhla, Thailand; ton331@hotmail.com; 5 Department of Biotechnology and Laboratory Science in Medicine, National Yang Ming Chiao Tung University, Taipei 112304, Taiwan; jasonhuang@nycu.edu.tw; 6 Food Technology and Innovation Research Center of Excellence, Research and Innovation Institute of Excellence, Walailak University, Tha Sala 80161, Nakhon Si Thammarat, Thailand

**Keywords:** viniferin, hepatocellular carcinoma, ADMET, network pharmacology, molecular docking, molecular dynamics simulation

## Abstract

**Background/Objectives:** Hepatocellular carcinoma (HCC) is a primary malignancy often driven by metabolic syndrome, fatty liver disease, and chronic hepatitis. These conditions foster a pro-inflammatory microenvironment that promotes tumor progression. Viniferin, a natural oligostilbene, has gained attention for its potential bioactivity. This study utilized an in silico network pharmacology approach to elucidate the pharmacokinetic properties and molecular mechanisms of ε- and δ-viniferin against HCC within the context of metabolic and inflammatory liver pathologies. **Methods:** ADMET profiles were characterized using SwissADME and pkCSM. Therapeutic targets were identified by intersecting viniferin-associated molecules with disease genes from GeneCards. A protein–protein interaction (PPI) network was constructed, supplemented by GO and KEGG enrichment analyses. Molecular docking and 200 ns of molecular dynamics (MD) simulations evaluated the binding affinity and structural stability between viniferin isomers and identified hub proteins. **Results:** Both ε- and δ-viniferin showed favorable drug-like properties, including high gastrointestinal absorption and low hepatotoxicity. We identified 247 overlapping targets, with network analysis highlighting ten essential hub genes, including *AKT1*, *HSP90AA1*, *ESR1*, *HIF1A*, *NFKB1*, *GSK3B*, *PTGS2*, *APP*, *MTOR*, and *PIK3CA*. Enrichment analysis confirmed their involvement in critical oncogenic pathways. Molecular docking showed strong interactions with APP, HSP90AA1, and AKT1, while MD simulations validated the long-term stability of ε-viniferin within the APP binding pocket. **Conclusions:** These findings provide mechanistic insights into viniferin as a multi-target agent for HCC, justifying further experimental validation in pre-clinical models.

## 1. Introduction

Hepatocellular carcinoma (HCC) is the sixth most common cancer worldwide, ranking fifth among men and ninth among women. Its mortality has increased steadily, with more than 866,000 new cases and an age-standardized rate (ASR) of 8.6 per 100,000 reported in 2022 [1]. The incidence of HCC is particularly high in Asia and Africa and is strongly associated with chronic hepatitis B and C infections, alcohol consumption, and metabolic dysfunction [2]. In Thailand, liver cancer represents one of the most prevalent malignancies and a major cause of cancer-related death [3]. Globally, the burden of HCC continues to rise, driven by alcohol use, viral infections, obesity, diabetes, and cirrhosis. Most cases arise from chronic liver injury, particularly metabolic dysfunction-associated steatotic liver disease (MASLD), which can progress to cirrhosis and markedly increase the risk of malignant transformation [4]. Additional etiologies include aflatoxin exposure, toxic chemicals such as vinyl chloride, and genetic disorders, including hemochromatosis, all of which contribute to hepatocyte damage and oncogenic mutations [5].

Metabolic syndrome and obesity have emerged as major drivers of chronic liver disease worldwide. MASLD affects approximately 32% of adults globally, with prevalence exceeding 40% in Southeast Asia, largely due to its strong association with insulin resistance and obesity [6]. Alcohol-associated fatty liver disease (AFLD) likewise contributes substantially to liver-related morbidity and mortality, with approximately 2.6 million deaths attributed to alcohol consumption in 2019 [7]. Importantly, metabolic syndrome, MASLD, and AFLD share persistent hepatic inflammation as a central pathogenic mechanism that promotes hepatocyte injury, fibrosis, and progression to cirrhosis [6]. In addition, viral hepatitis remains a major global health concern, with hepatitis B virus (HBV) and hepatitis C virus (HCV) accounting for more than 300 million chronic infections worldwide [8,9]. The convergence of metabolic dysfunction, chronic viral infection, and sustained inflammatory responses significantly increases the risk of HCC development, highlighting the need for improved molecular-level understanding of hepatocarcinogenesis.

At the molecular level, HCC develops through a multistep process characterized by chronic inflammation, metabolic dysregulation, and genetic alterations. Persistent liver injury triggers the release of damage-associated molecular patterns (DAMPs) and pathogen-associated molecular patterns (PAMPs), activating innate immune signaling and promoting fibrogenesis and tumorigenesis [10]. Dysregulation of key signaling pathways such as PI3K/AKT/mTOR, MAPK, JAK/STAT, and Wnt/β-catenin contributes to hepatocyte proliferation, metabolic imbalance, and resistance to apoptosis during hepatocarcinogenesis [11,12,13,14]. In addition, genomic instability associated with viral hepatitis further accelerates tumor progression through activation of oncogenic pathways and disruption of cell-cycle regulation [15]. As HCC progresses, angiogenesis promotes tumor growth, and vascular invasion facilitates metastasis. Although surgical resection remains a primary treatment, recurrence rates are high [16]. Current systemic therapies, including sorafenib and cytotoxic chemotherapy, provide limited survival benefits due to drug resistance and impaired hepatic function [17,18]. While immune checkpoint inhibitors such as anti-PD-1 antibodies have emerged as promising therapeutic options [19], the modest efficacy and potential toxicity of existing treatments have increased interest in plant-derived phytochemicals that can modulate cancer-related pathways with lower toxicity.

**Figure 1:**
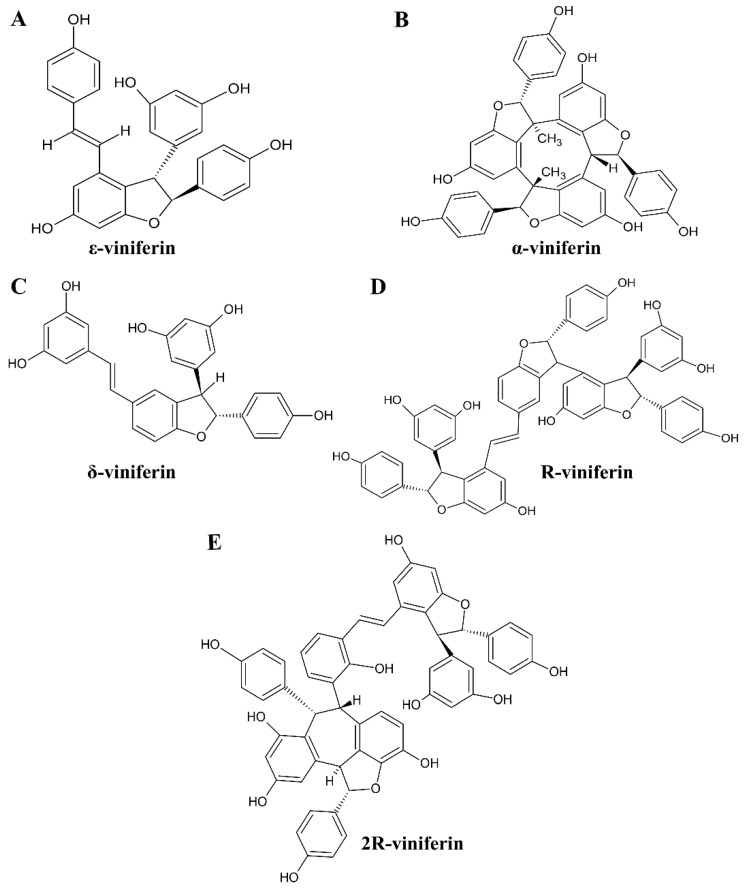
Chemical structures of viniferin derivatives, including ε-viniferin (**A**), α-viniferin (**B**), δ-viniferin (**C**), R-viniferin (**D**), and 2R-viniferin (**E**). Molecular structures were obtained from the PubChem database and redrawn using ChemSketch for clarity.

Viniferin, a resveratrol dimer found in *Gnetum gnemon* and *Vitis vinifera*, exists in several structural forms, including α-, ε-, δ-, R-, and 2R-viniferin [20]. The chemical structures of these viniferin derivatives are shown in Figure 1. As members of the stilbene family, viniferin derivatives can exist in both cis and trans configurations, similar to their precursor resveratrol, and both forms of ε- and δ-viniferin have been reported in the literature [21]. Cis-viniferin and trans-viniferin are geometric isomers of a resveratrol dimer, differing in the spatial arrangement of their double bonds, which directly influences their stability and bioactivity. The key distinction lies in their configuration: trans-viniferin has functional groups on opposite sides of the double bond (Figure 1A) and is generally more stable with stronger anti-inflammatory and antioxidant properties, whereas cis-viniferin has them on the same side, making it less stable, light-sensitive, and prone to increased concentration upon exposure to light. Viniferin exhibits notable anticancer activity through induction of apoptosis and modulation of Bax/Bcl-2 signaling [22,23]. In HCC models, 2R-viniferin suppresses cell proliferation and induces G2/M cell-cycle arrest in HepG2 cells [23], while apoptotic effects have also been reported for ε-viniferin in combination with vincristine [24], including nanoparticle-based delivery systems [25]. In addition, α- and ε-viniferin induce reactive oxygen species (ROS)-dependent mitochondrial dysfunction and G2/M arrest [26], and α-viniferin exhibits anti-angiogenic activity by inhibiting the VEGFR-2/p70S6K pathway [27]. Beyond cancer, viniferin regulates inflammatory signaling via STAT-1 [16], exhibits anti-obesity activity through suppression of PPAR-γ in high-fat diet-induced mice [28], and confers protective effects in type 2 diabetes [20]. Notably, the combination of trans-resveratrol and trans-ε-viniferin demonstrates hepatoprotective effects by reducing lipid peroxidation and suppressing pro-inflammatory mediators such as TNF-α and COX-2 [29]. Despite these promising activities, viniferin’s poor bioavailability necessitates integrative strategies to elucidate its systemic mechanisms of action.

Modern drug discovery increasingly relies on computer-aided drug design (CADD) to improve efficiency and reduce development costs. Early-stage pharmacokinetic prediction of pharmacokinetic and ADMET properties enables prioritization of compounds with favorable drug-like characteristics [30,31]. Network pharmacology has emerged as a systems-level approach to address the limitations of the traditional “one drug–one target” paradigm, particularly in complex diseases such as cancer [32]. Through polypharmacology mapping, multi-target compounds can be identified and evaluated within biological networks [33]. In addition, molecular docking and molecular dynamics (MD) simulations are widely used to predict protein–ligand interactions and evaluate the stability of these interactions under dynamic conditions [34,35]. In this study, we investigate the mechanisms of viniferin in metabolic syndrome, fatty liver disease, and liver cancer using an integrated workflow combining network pharmacology, molecular docking, and MD simulations, intending to support its future development as a targeted therapeutic strategy.

## 2. Materials and Methods

### 2.1. Analysis of Chemical Properties and Drug-Likeness

The physicochemical properties and drug-likeness profiles of ε-viniferin, α-viniferin, δ-viniferin, R-viniferin, and 2R-viniferin were systematically evaluated using the SwissADME platform (http://www.swissadme.ch; accessed on 5 May 2025). SwissADME provides comprehensive chemoinformatics analyses, including physicochemical descriptors and pharmacokinetic predictions relevant to oral drug development. Drug-likeness assessment was guided by Lipinski’s Rule of Five (RO5), which serves as an initial screening criterion for compounds with favorable oral bioavailability. According to RO5, compounds are more likely to be orally active if they meet the following criteria: molecular weight ≤ 500 g/mol, lipophilicity (logP) ≤ 5, topological polar surface area (TPSA) ≤ 140 Å^2^, no more than 10 hydrogen bond acceptors, and no more than 5 hydrogen bond donors. These parameters collectively reflect the balance between solubility and membrane permeability required for effective absorption and systemic distribution [36].

### 2.2. Pharmacokinetics and Toxicity Prediction

Pharmacokinetic behavior and toxicity profiles of viniferin derivatives were evaluated using the pkCSM ADMET database (http://biosig.unimelb.edu.au/pkcsm/prediction; accessed on 5 May 2025). pkCSM is a computational platform that predicts absorption, distribution, metabolism, excretion, and toxicity (ADMET) properties based on graph-based molecular structural signatures. This analysis provided quantitative predictions for key biological performance indicators, including aqueous solubility, gastrointestinal absorption, skin permeability, and distribution characteristics, particularly blood–brain barrier penetration. In addition, metabolic interactions with hepatic cytochrome P450 enzymes, renal clearance parameters (including OCT2 substrate prediction), and toxicological endpoints—such as AMES mutagenicity, hepatotoxicity, and hERG channel inhibition—were assessed to evaluate overall safety and clinical feasibility [37].

### 2.3. Selection of Molecular Targets and Disease-Related Genes

The chemical structures of viniferin isomers were retrieved from the PubChem database (https://pubchem.ncbi.nlm.nih.gov/; accessed on 5 May 2025) in standard SMILES format. Target prediction was performed using the SwissTargetPrediction web server (http://www.swisstargetprediction.ch/; accessed on 5 May 2025), which integrates two-dimensional chemical similarity and three-dimensional pharmacophore modeling to identify probable human protein targets. Additional target predictions were obtained from the SEA database (https://sea.bkslab.org/; accessed on 5 May 2025) and SuperPred (https://prediction.charite.de/; accessed on 5 May 2025). All predicted targets were standardized by mapping to UniProt identifiers (http://www.uniprot.org/; accessed on 5 May 2025), including official gene names and protein symbols. Disease-associated gene datasets related to metabolic syndrome, fatty liver disease, chronic hepatitis, and liver cancer were retrieved from the GeneCards database (https://www.genecards.org/; accessed on 5 May 2025). Selection was restricted to Homo sapiens genes with high relevance scores. Predicted viniferin targets were intersected with disease-related gene sets using the InteractiVenn tool (https://www.interactivenn.net/; accessed on 5 May 2025) to identify overlapping genes representing potential therapeutic intervention sites [38].

### 2.4. GO and KEGG Pathway Enrichment Analysis

To investigate the functional relevance of the identified target genes, enrichment analyses were performed using the Gene Ontology (GO) and Kyoto Encyclopedia of Genes and Genomes (KEGG) databases through the ShinyGO 0.82 platform (http://bioinformatics.sdstate.edu/go/; accessed on 5 May 2025). All analyses were restricted to *H. sapiens* genes. Statistical significance was defined as *p* < 0.05 with a false discovery rate (FDR) < 0.05 to control multiple testing. GO enrichment analysis focused on the top 20 significantly enriched terms across three categories: molecular function (MF), biological process (BP), and cellular component (CC).

To further elucidate the mechanistic relevance of viniferin, KEGG pathway enrichment analysis was conducted using the same statistical thresholds and methodological parameters applied in the GO analysis to ensure consistency across datasets. This analysis quantified the number of target genes mapped to each significantly enriched pathway, thereby identifying molecular signaling circuits potentially modulated by viniferin in liver cancer-related networks. Integration of GO and KEGG results provided a comprehensive overview of both functional annotations and disease-associated pathways, offering insight into the pharmacological mechanisms of viniferin at the systems level [39].

### 2.5. Protein–Protein Interaction (PPI) Network Construction

To systematically analyze the molecular interactions among viniferin-associated targets, a protein–protein interaction (PPI) network was constructed using the STRING database (https://www.string-db.org/; accessed on 5 May 2025). The analysis was limited to *H. sapiens* proteins, and only interactions with a medium or higher confidence score (>0.4) were included to ensure network reliability. The resulting PPI network was visualized and analyzed using Cytoscape software (version 3.10.3; Institute for Systems Biology, Seattle, WA, USA). To identify proteins with central regulatory roles, the cytoHubba plugin was applied to calculate topological parameters and rank nodes based on degree centrality. Proteins with the highest connectivity were defined as hub proteins and considered critical mediators of viniferin’s pharmacological effects [40].

### 2.6. Calculation of Target Protein–Ligand Interactions Using Molecular Docking

Molecular docking simulations were performed to characterize binding interactions and drug–target recognition between viniferin isomers and the top 10 identified hub proteins. Three-dimensional structures of viniferin isomers were optimized using UCSF Chimera (version 1.17.1; University of California, San Francisco, CA, USA) by adding missing hydrogen atoms at physiological pH (7.4) and assigning Gasteiger charges. Energy minimization was conducted using the steepest descent algorithm.

Crystal structures of target proteins were retrieved from the Protein Data Bank (https://www.rcsb.org/; accessed on 13 June 2025). Protein preparation was carried out using BIOVIA Discovery Studio 2025, including removal of water molecules and co-crystallized ligands. Kollman united-atom charges were assigned using AutoDock Tools (ADT), and prepared structures were saved in PDBQT format. Docking simulations were executed using AutoDock4 (version 4.2; BIOVIA, San Diego, CA, USA) with the Lamarckian genetic algorithm. Each docking protocol consisted of 50 independent runs with a population size of 200, using a grid box encompassing the active site of each protein. Binding affinities (ΔG) and interaction energies were calculated and docking poses were visualized and analyzed using Discovery Studio 2025 [41].

### 2.7. Molecular Dynamics (MD) Simulations

To assess the dynamic stability and structural integrity of the ligand–protein complexes, molecular dynamics (MD) simulations were conducted for 200 nanoseconds (ns) and compared with reference drug–protein complexes. The simulations were performed in an aqueous environment at pH 7.0 using the TIP3P water model. Systems were solvated in an orthorhombic box (10 × 10 × 10 Å) and neutralized with 0.15 M NaCl to mimic physiological ionic strength. MD simulations were carried out under an NPT ensemble at 310 K and 1.01 bar. Long-range electrostatic interactions were treated using the Smooth Particle Mesh Ewald (PME) method. Post-simulation analyses included calculation of root mean square deviation (RMSD) and root mean square fluctuation (RMSF) for both protein backbones and ligands, as well as generation of ligand–protein interaction maps to evaluate long-term binding stability and key residue interactions throughout the simulation period [42].

## 3. Results

### 3.1. Chemoinformatics and Drug Likeness Properties of Viniferin Derivatives

The SWISS ADME analysis of viniferin derivatives revealed substantial differences in their physicochemical properties and drug-likeness profiles, highlighting variability in their potential as therapeutic candidates as shown in Table 1. ε- and δ-Viniferin emerged as the most promising molecules, with molecular weights of 454.47 g/mol, values well within the acceptable threshold (<500 g/mol), and moderate lipophilicity. Although TPSA values of 195.45 Å slightly exceeded the recommended cut-off (<140 Å), both compounds maintained acceptable numbers of hydrogen bond donors and acceptors. Importantly, they satisfied multiple drug-likeness filters, including Lipinski, Veber, and Egan rules, suggesting favorable oral bioavailability and permeability. These characteristics position ε- and δ-viniferin as suitable candidates for further pharmacological evaluation and potential drug development.

In contrast, α-viniferin, R-viniferin, and 2-R-viniferin showed lower compliance with conventional drug-likeness criteria. α-Viniferin exhibited a molecular weight of 678.69 g/mol and a LogP of 8.03, both exceeding recommended limits, alongside 9 hydrogen bond acceptors and 6 donors, which further diminished its drug-like potential. R-viniferin and 2-R-viniferin were even less favorable, with extremely high molecular weights, excessive lipophilicity (LogP > 10), and elevated hydrogen bonding capacity. Their TPSA values, approximately 388 Å, were far above the threshold, indicating poor permeability and limited bioavailability. These compounds also failed to meet Lipinski, Veber, and Egan criteria, reinforcing their classification as non-drug-like molecules unsuitable for further consideration. However, it should be noted that drug-likeness rules such as Lipinski, Veber, and Egan have limited ability to distinguish structurally similar isomeric compounds. Because viniferin derivatives share highly similar molecular frameworks, many calculated physicochemical parameters show minimal variation among the isomers. Therefore, these rules were used primarily as preliminary indicators of drug-likeness rather than strict criteria for compound exclusion.

### 3.2. In Silico Pharmacokinetic and Toxicological Assessment of Stilbene Derivatives

The pharmacokinetic analysis of ε-viniferin and δ-viniferin as summarized in Table 2 using the PkCSM program revealed that both compounds exhibit poor water solubility and low Caco2 permeability, though ε-viniferin showed higher intestinal absorption (96.06%) compared to δ-viniferin (87.91%). Both are substrates and inhibitors of P-glycoprotein I and II, with similar skin permeability values. Distribution profiles indicated low VDss values and limited blood–brain barrier penetration, with δ-viniferin showing slightly lower BBB permeability. In metabolism, both act as CYP3A4 substrates and CYP2C9 inhibitors, but only δ-viniferin inhibits CYP2C19. Clearance rates were low for both, and neither is a renal OCT2 substrate. Toxicity predictions showed no AMES toxicity, hepatotoxicity, or skin sensitization, with both compounds inhibiting hERG II but not hERG I. Acute and chronic oral rat toxicity values were comparable, while δ-viniferin demonstrated slightly higher chronic toxicity. Environmental toxicity assessments revealed identical *T. pyriformis* toxicity but greater minnow toxicity for ε-viniferin. Overall, the two stilbenes share similar pharmacokinetic and toxicity profiles, with minor differences in absorption, metabolism, and environmental toxicity.

### 3.3. Identification of Viniferin Targets and Disease-Related Genes

**Figure 2:**
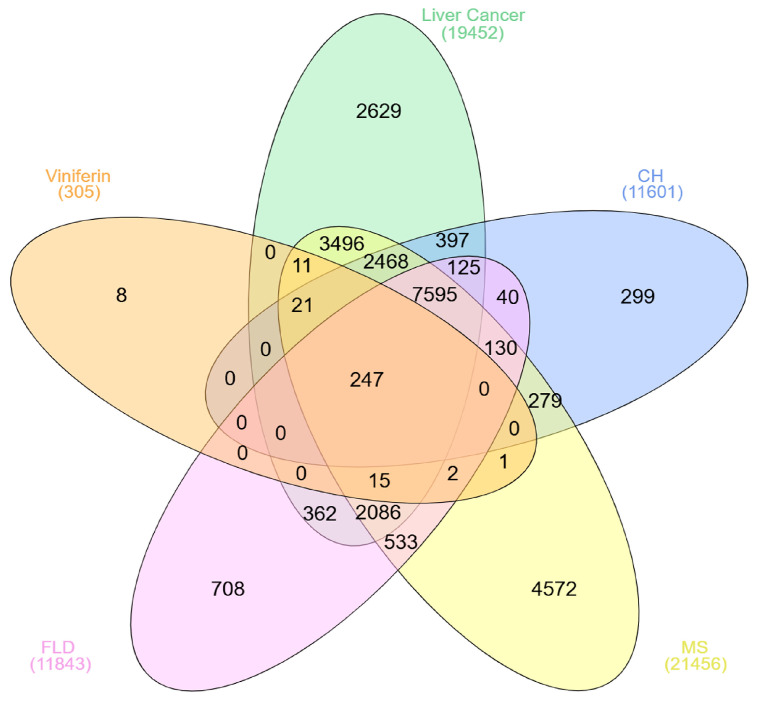
Overlapping gene relationships of viniferin with metabolic syndrome (MS), fatty liver disease (FLD), chronic hepatitis (CH), and liver cancer, presented in a Venn diagram.

The Venn diagram analysis (Figure 2) revealed that viniferin is associated with a broad set of 305 genes, of which 247 are commonly shared across metabolic syndrome (MS), fatty liver disease (FLD), chronic hepatitis (CH), and liver cancer or HCC, highlighting a strong genetic overlap among these conditions. Beyond the central intersection, viniferin also shares substantial subsets of genes with individual diseases and their pairwise or multi-disease overlaps, reflecting its potential pleiotropic influence on liver-related pathologies. The largest overlaps were observed between viniferin and MS (21,456 genes) and FLD (11,843 genes), followed by CH (11,601 genes) and liver cancer (19,452 genes), suggesting that viniferin may interact with key molecular pathways implicated in metabolic and hepatic disorders. Overall, the results emphasize that viniferin targets a core group of genes relevant to multiple liver diseases, supporting its potential as a multi-target therapeutic candidate for metabolic and hepatic syndromes, including HCC.

### 3.4. Identification of Viniferin Targets and Viniferin-Disease Target Network Construction

**Figure 3:**
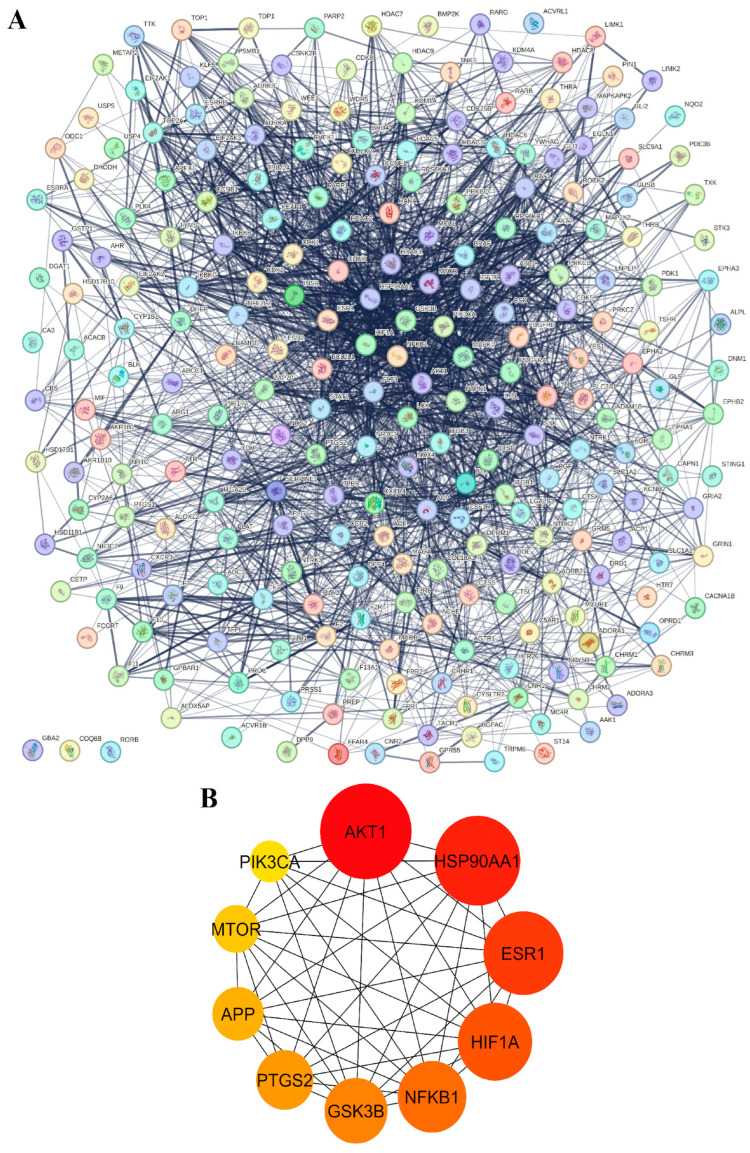
Protein–protein interaction (PPI) networks of candidate targets. (**A**) Overall PPI network showing interactions among potential target proteins, with nodes representing proteins and edges indicating validated or predicted connections. (**B**) PPI network of 10 key targets, where node color intensity (yellow to red) reflects relative connectivity and importance.

Subsequently, a protein–protein interaction (PPI) network analysis was performed using the STRING database, with the results shown in Figure 3. In the PPI network, circular nodes represent proteins, within which their three-dimensional structures are displayed. The connecting lines between nodes indicate protein interactions, with stronger interactions represented by a greater number and thickness of lines. In addition, the top 10 hub genes with the highest degrees of interaction were calculated and identified using the cytoHubba plug-in in Cytoscape version 3.10.3, applying topological analysis based on the degree parameter. These hub genes were selected as key regulatory genes for the treatment of MS, FLD, CH, and HCC (Figure 3A). Nodes with darker red coloration indicate higher degree scores. The major hub target proteins identified in this analysis were *AKT1*, *HSP90AA1*, *ESR1*, *HIF1A*, *NFKB1*, *GSK3B*, *PTGS2*, *APP*, *MTOR*, and *PIK3CA*, as shown in Figure 3B. These hub genes were therefore selected as key regulatory targets for metabolic syndrome, fatty liver disease, chronic hepatitis, and liver cancer, underscoring their critical roles in mediating viniferin’s biological activity in pathways related to inflammation, cell survival, metabolism, and stress responses. Overall, the PPI results emphasize that viniferin acts through a multi-target mechanism by engaging key signaling proteins that may contribute to its therapeutic potential, and these targets were subsequently selected for mechanistic investigation using molecular docking approaches.

### 3.5. Functional Analysis of Target Genes Using GO Enrichment Analysis

**Figure 4:**
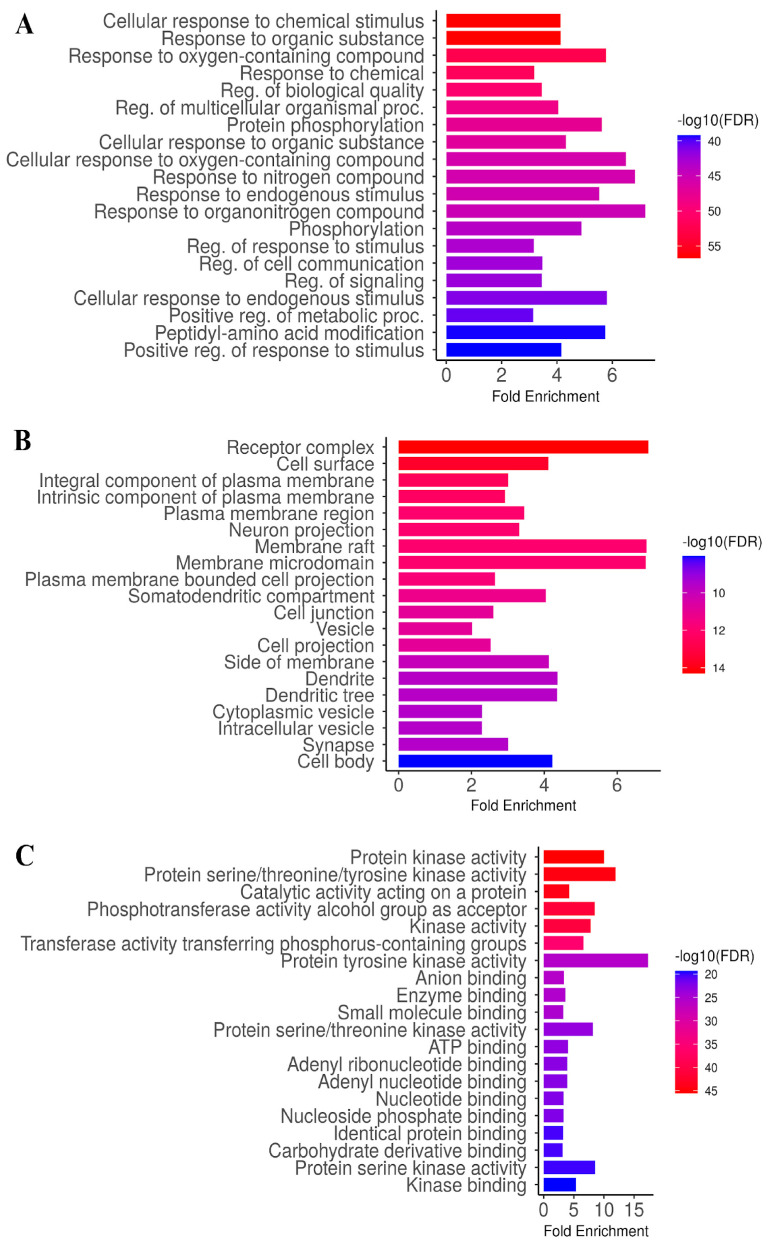
GO enrichment analysis of viniferin in the treatment of metabolic syndrome, fatty liver, chronic hepatitis, and liver cancer (*p* < 0.05), including analyses of Biological Process (**A**), Cellular Component (**B**), and Molecular Function (**C**).

To elucidate the biological significance of the 247 target genes identified from the Venn diagram, enrichment analysis was performed using ShinyGO 0.82. The results highlighted a substantial number of genes with false discovery rate (FDR) or *p*-values below 0.05. Specifically, 2863 genes were enriched in biological processes, 1033 genes were associated with cellular components, and 1463 genes were linked to molecular functions, as illustrated in Figure 4A–C. The GO enrichment analysis of viniferin revealed broad biological involvement across processes, cellular components, and molecular functions relevant to metabolic syndrome, fatty liver disease, chronic hepatitis, and liver cancer. In the GO biological process (GOBP) category, enriched terms included cellular responses to chemical and oxygen-containing compounds, regulation of cell communication and proliferation, phosphorylation, and positive regulation of metabolic processes, highlighting viniferin’s role in modulating stress responses and signaling cascades (Figure 4A). The GO cellular component (GOCC) analysis emphasized enrichment in receptor complexes, plasma membrane regions, cell junctions, vesicles, and synaptic structures, suggesting that viniferin targets are localized to key sites of signal transduction and intercellular communication (Figure 4B). Finally, the GO molecular function (GOMF) category was dominated by kinase-related activities, including protein kinase, serine/threonine/tyrosine kinase, and phosphotransferase functions, alongside ATP and nucleotide binding, underscoring viniferin’s potential to regulate phosphorylation-dependent signaling pathways (Figure 4C). Collectively, these results indicate that viniferin exerts multifaceted effects by modulating cellular responses, membrane-associated complexes, and kinase-driven molecular mechanisms central to disease progression.

### 3.6. Functional Analysis of Target Genes Using KEGG Enrichment Analysis

**Figure 5:**
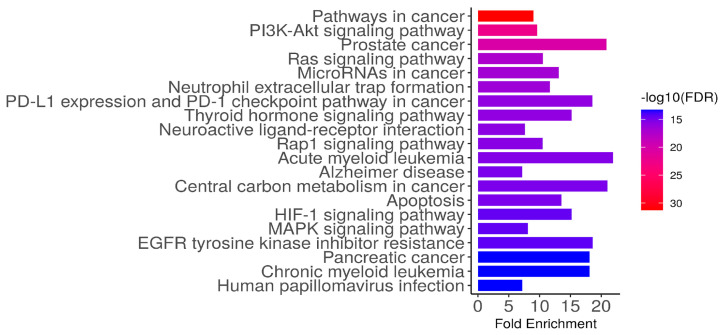
Mechanistic analysis using KEGG enrichment of the 247 identified candidate targets (*p* < 0.05), highlighting potential target genes involved in MS, FLD, CH, and HCC.

The KEGG enrichment analysis of the 247 identified candidate targets of viniferin revealed significant involvement in multiple cancer-related and signaling pathways, underscoring their potential mechanistic roles in metabolic syndrome, fatty liver disease, chronic hepatitis, and liver cancer (Figure 5). A total of 410 pathways (FDR < 0.05) were identified as significantly enriched. Highly represented pathways included pathways in cancer, PI3K–Akt signaling, Ras signaling, and MAPK signaling, alongside disease-specific pathways such as prostate cancer, pancreatic cancer, acute and chronic myeloid leukemia, and Alzheimer’s disease. Immune-related processes, including PD-L1/PD-1 checkpoint regulation and neutrophil extracellular trap formation, were also prominent, while metabolic and stress-response pathways such as apoptosis, HIF-1 signaling, and central carbon metabolism in cancer further highlighted the diverse biological functions of these targets. Collectively, these findings suggest that the candidate genes are broadly implicated in oncogenesis, immune regulation, and metabolic dysregulation, providing mechanistic insight into their relevance in liver-associated diseases.

### 3.7. Confirmation of Hub Targets by Molecular Docking

To validate the inhibitory potential of viniferin against liver cancer-associated proteins, molecular docking studies were performed on ten selected targets. Binding interactions were evaluated based on docking energy values, with affinity interpreted in a tiered manner. Complexes exhibiting binding energies of ≤−10.0 kcal/mol were classified as strong binders, those between −10.0 and −8.0 kcal/mol as moderate binders, and values > −8.0 kcal/mol as weak binders. The results revealed that both ε- and δ-viniferin showed measurable binding interactions with ESR1, HIF1A, NFKB1, GSK3B, PTGS2, MTOR, and PIK3CA. However, the predicted binding energies did not reach the predefined threshold of strong binding affinity (≤−10 kcal/mol). These results therefore suggest weak to moderate binding interactions rather than complete binding interaction. In contrast, significant binding affinity was observed against three hub proteins, APP, AKT1, and HSP90AA1, suggesting that viniferin selectively targets key regulators involved in liver cancer progression (Table 3).

**Figure 6:**
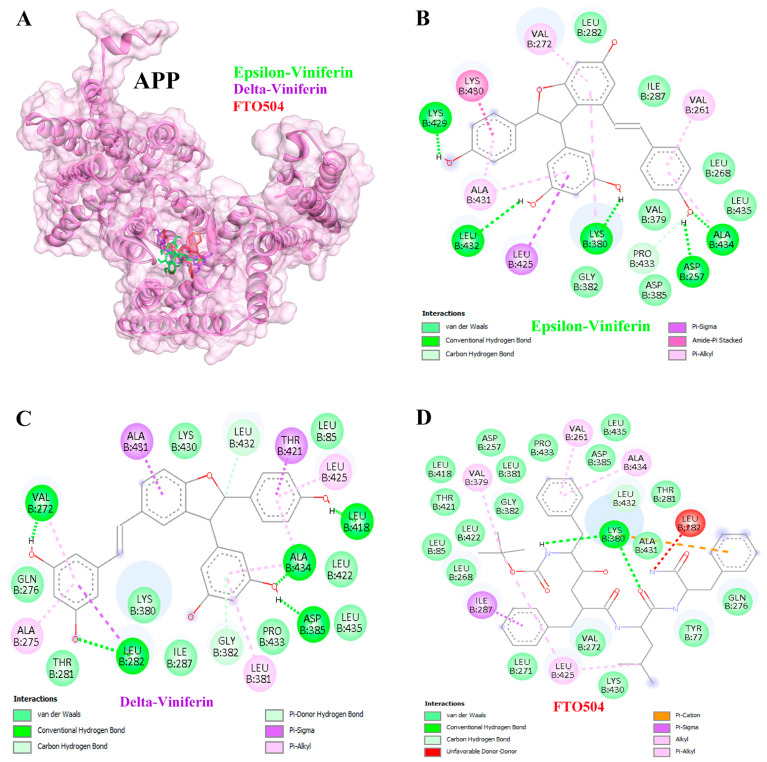
Binding interactions between ligands and the APP target protein (**A**). Molecular docking studies of epsilon (ε)-viniferin (**B**) and delta (δ)-viniferin (**C**) compared with the positive control compound FTO504 (**D**) on the APP target protein. Colored dashed lines indicate different types of ligand–protein interactions, including hydrogen bonds and hydrophobic interactions.

**Figure 7:**
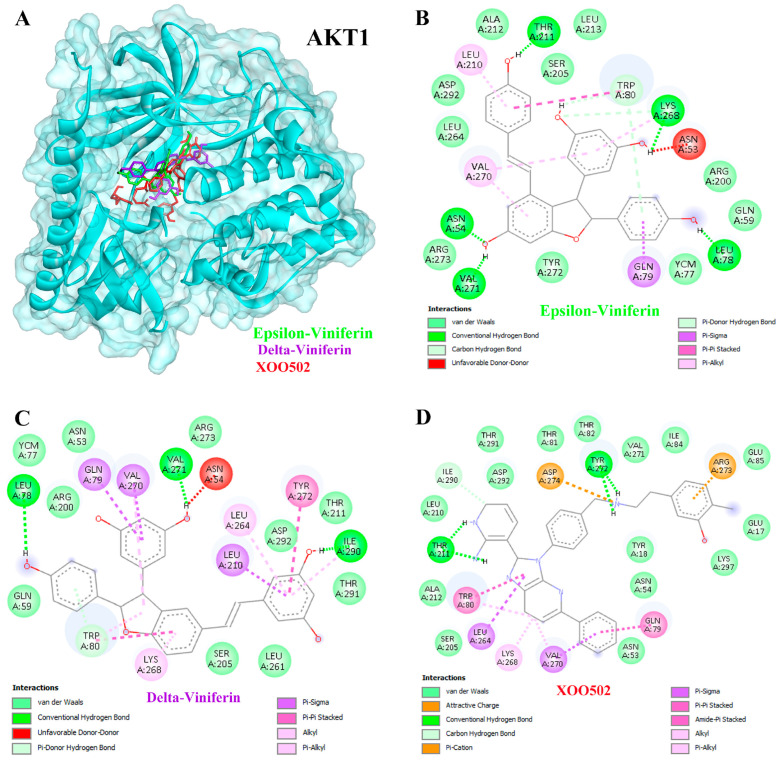
Binding interactions between ligands and the AKT1 target protein (**A**). Molecular docking studies of epsilon (ε)-viniferin (**B**) and delta (δ)-viniferin (**C**) compared with the positive control compound XOO502 (**D**) on the AKT1 target protein. Colored dashed lines indicate different types of ligand–protein interactions, including hydrogen bonds and hydrophobic interactions.

Docking analysis demonstrated that ε- and δ-viniferin inhibited APP more effectively than the positive control FTO504. ε-Viniferin exhibited a binding energy of −10.01 kcal/mol with an inhibition constant of 46.34 nM, while δ-viniferin showed superior binding at −11.12 kcal/mol with an inhibition constant of 7.11 nM (Table 3). Hydrogen bond interactions were observed at residues LYS429, LEU432, LYS380, ASP257, and ALA434 for ε-viniferin (Figure 6A,B), and at VAL272, LEU282, LEU418, ALA434, and ASP385 for δ-viniferin (Figure 6A,C). Similarly, both compounds inhibited AKT1 more effectively than the positive control XOO502. ε-Viniferin displayed a binding energy of −10.44 kcal/mol (22.22 nM), while δ-viniferin achieved −10.86 kcal/mol (10.91 nM). Key hydrogen bond interactions included THR211, LYS268, LEU78, VAL271, and ASN54 for ε-viniferin (Figure 7A,B), and LEU78, VAL271, and ILE290 for δ-viniferin (Figure 7A,C).

**Figure 8:**
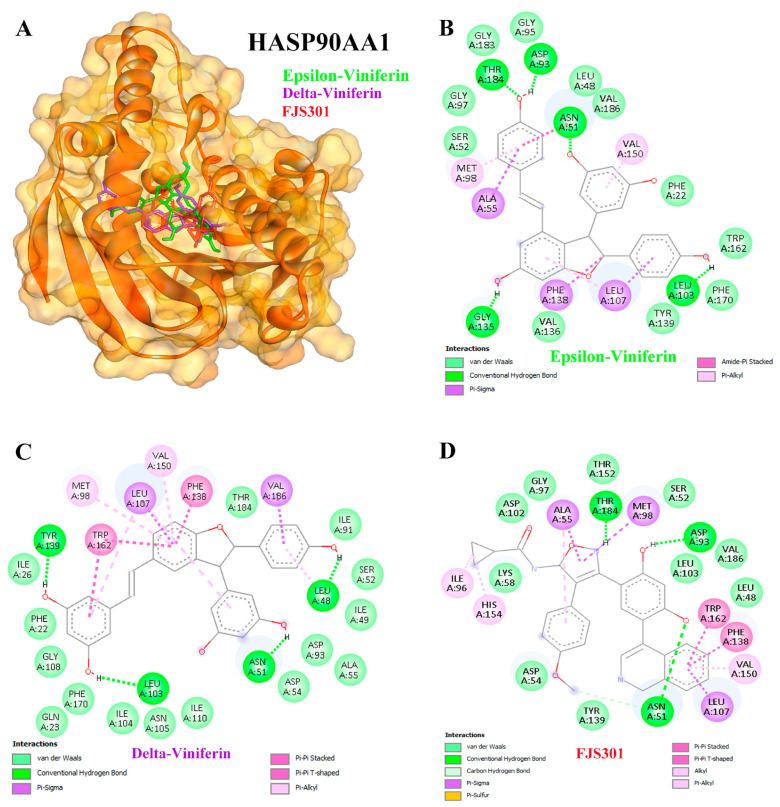
Binding interactions between ligands and the HASP90AA1 target protein (**A**). Molecular docking studies of epsilon (ε)-viniferin (**B**) and delta (δ)-viniferin (**C**) compared with the positive control compound FJS301 (**D**) on the HASP90AA1 target protein. Colored dashed lines indicate different types of ligand–protein interactions, including hydrogen bonds and hydrophobic interactions.

Viniferin also showed strong inhibitory activity against HSP90AA1 compared to the positive control FJS301. ε-Viniferin exhibited a binding energy of −10.39 kcal/mol with an inhibition constant of 24.05 nM, while δ-viniferin demonstrated the most potent binding at −11.41 kcal/mol with an inhibition constant of 4.33 nM (Table 3). Hydrogen bond interactions for ε-viniferin were observed at THR184, ASP93, ASN51, GLY135, and LEU103 (Figure 8A,B), whereas δ-viniferin formed interactions at TYR139, LEU103, ASN51, and LEU48 (Figure 8A,C). Collectively, these findings highlight the selective binding potential of viniferin, particularly δ-viniferin, against APP, AKT1, and HSP90AA1, supporting its role as a promising candidate for liver cancer therapy.

### 3.8. Binding Ability of Viniferin with APP Target Protein Using MD Simulation

Preliminary analysis from molecular docking showed that viniferin has high potential as an active compound, as it strongly binds to key amino acids within the active site of the APP and achieves significantly better binding scores compared to the positive control drug. To confirm the stability of this binding, molecular dynamics simulations were performed for 200 nanoseconds, with comparison to the known APP inhibitor FTO504. Analysis of the RMSD served as a key indicator for evaluating the structural stability of the ligand–protein complexes. High RMSD values generally reflect structural instability, whereas stable values indicate equilibrium and consistent binding.

**Figure 9:**
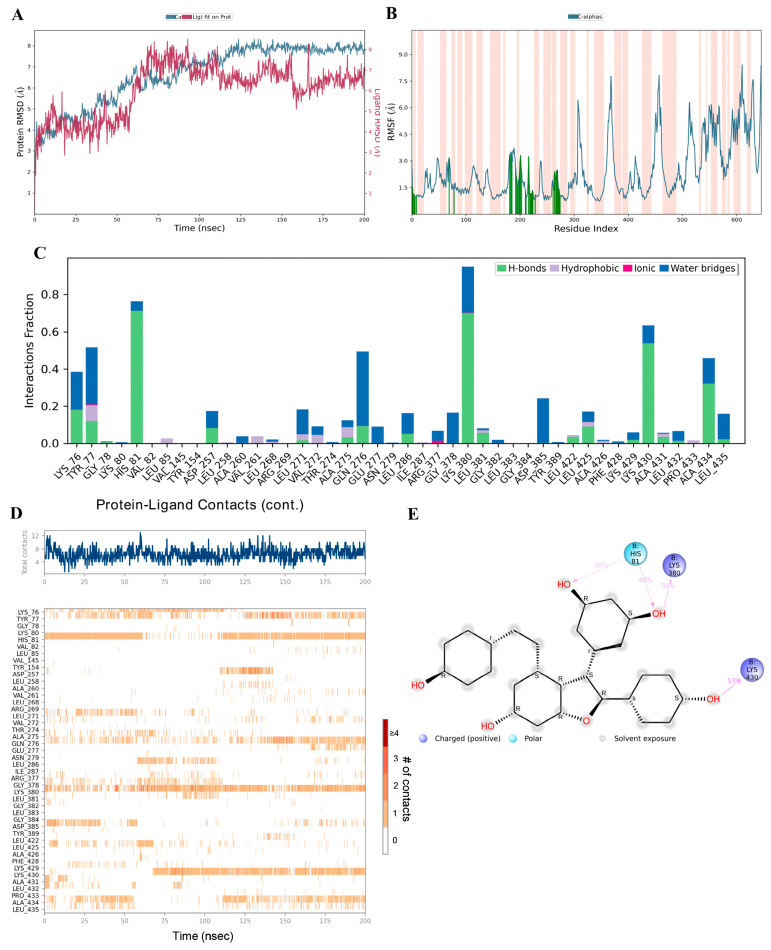
Binding capability of epsilon (ε)-viniferin with the APP analyzed by molecular dynamics simulation, showing RMSD (**A**), RMSF plot showing the fluctuation of Cα atoms across residues. Orange-shaded regions indicate binding-site residues. (**B**), protein–ligand contact plots (**C**) showing the frequency of ligand–residue contacts throughout the simulation, including hydrogen bonds (green), hydrophobic interactions (purple), ionic interactions (pink), and water bridges (blue), MD timeline interaction analysis (**D**), and 2D interaction diagram of epsilon (ε)-viniferin with the APP (**E**).

**Figure 10:**
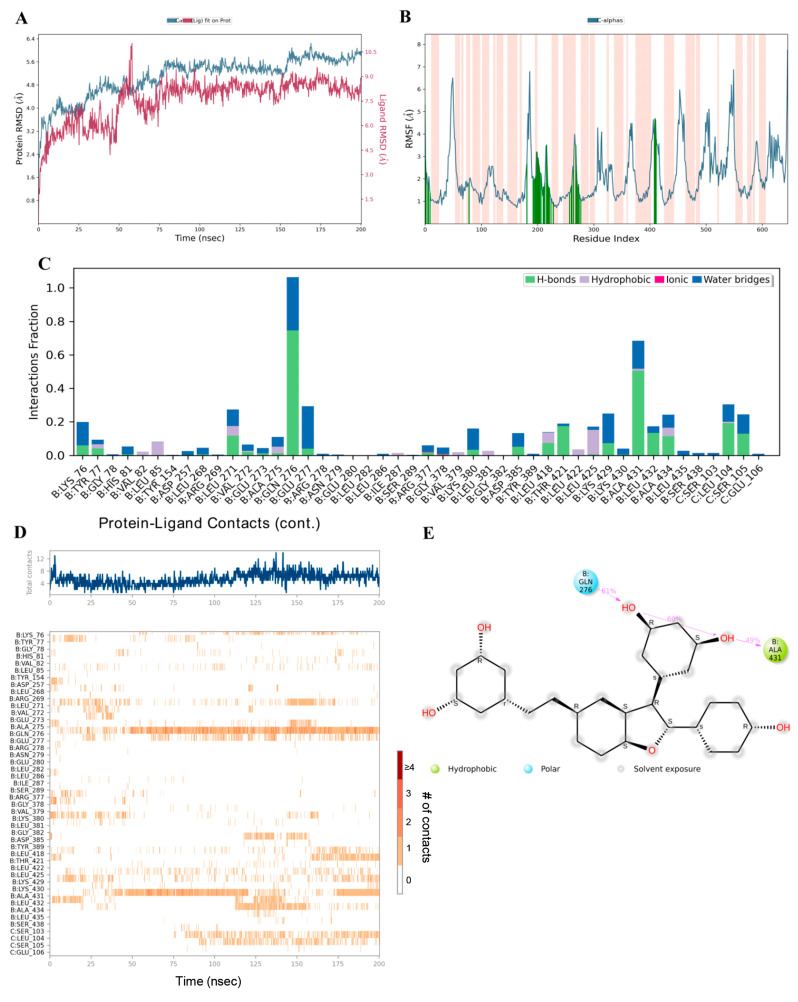
Binding capability of delta (δ)-viniferin with the APP analyzed by molecular dynamics simulation, showing RMSD (**A**), RMSF plot showing the fluctuation of Cα atoms across residues. Orange-shaded regions indicate binding-site residues (**B**), protein–ligand contact plots (**C**) showing the frequency of ligand–residue contacts throughout the simulation, including hydrogen bonds (green), hydrophobic interactions (purple), ionic interactions (pink), and water bridges (blue), MD timeline interaction analysis (**D**), and 2D interaction diagram of delta (δ)-viniferin with the APP (**E**).

**Figure 11:**
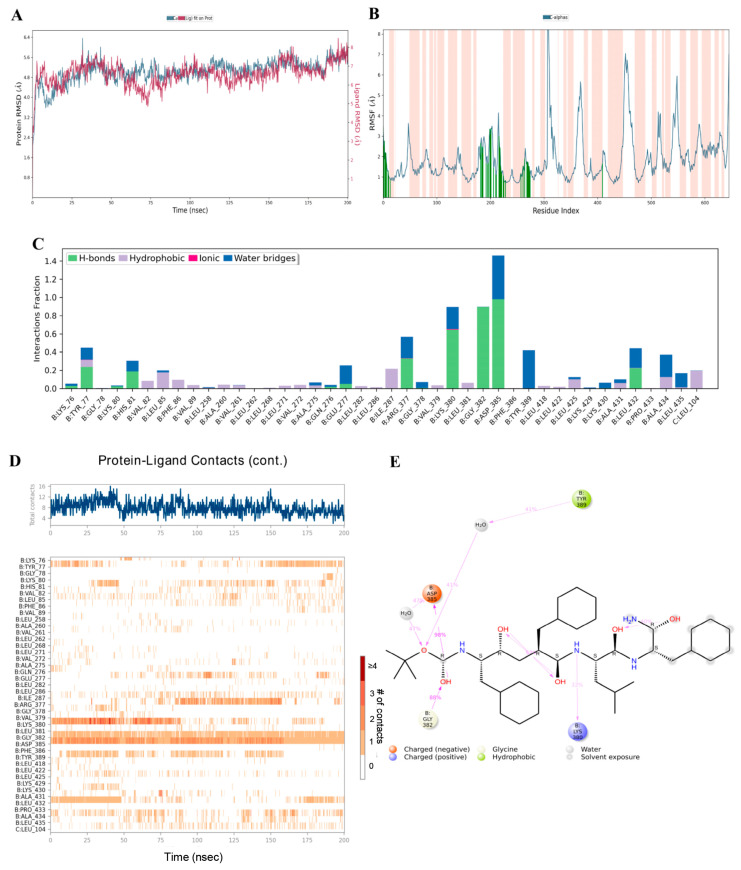
Binding capability of FTO504 with the APP analyzed by molecular dynamics simulation, showing RMSD (**A**), RMSF plot showing the fluctuation of Cα atoms across residues. Orange-shaded regions indicate binding-site residues. (**B**), protein–ligand contact plots (**C**) showing the frequency of ligand–residue contacts throughout the simulation, including hydrogen bonds (green), hydrophobic interactions (purple), ionic interactions (pink), and water bridges (blue), MD timeline interaction analysis (**D**), and 2D interaction diagram of FTO504 with the APP (**E**).

The simulations revealed that during the initial phase (0–70 ns), the RMSD values of ε-viniferin and δ-viniferin bound to APP were unstable, before reaching continuous stability until the end of the 200 ns simulation, reflecting structural equilibrium and stable binding (Figure 9A and Figure 10A). In contrast, FTO504 bound to APP showed stable RMSD values from the first 20 ns through to 200 ns (Figure 11A). Overall, all three systems demonstrated comparable structural stability.

RMSF analysis, which measures residue-specific flexibility, showed that ε- and δ-viniferin bound to APP at key positions similar to FTO504 (Figure 9B and Figure 10B). Most amino acids exhibited fluctuations below 3.0 Å, indicating limited atomic movement and supporting overall structural stability. Regions of higher flexibility were mainly observed in protein loop regions, while secondary structures such as α-helices and β-strands maintained their integrity throughout the simulation (Figure 9B and Figure 10B).

Further analysis of ligand–protein contact duration over 200 ns using MD timeline interaction analysis revealed that ε-viniferin maintained stable interactions with amino acids LYS76, TYR77, HIS81, LYS380, LYS430, and ALA434 (Figure 9C,D). δ-Viniferin showed stable interactions with GLN276 and ALA431 (Figure 10C,D), while FTO504 consistently bound to GLY382 and ASP385 throughout the simulation (Figure 11C,D).

Histogram analysis demonstrated that binding within the APP active site involved hydrogen bonds (green), hydrophobic interactions (purple), ionic interactions (red), and water-mediated linkages (blue). In the ε-viniferin–APP system, hydrogen bonds were mainly formed with HIS81, LYS380, LYS430, and ALA434 (Figure 9D). In the δ-viniferin–APP system, hydrogen bonds were formed with GLN276, THR421, ALA431, LEU432, and ALA434 (Figure 10D). In the FTO504–APP system, key hydrogen bonds were observed with TYR77, HIS81, ARG377, LYS380, GLY382, ASP385, and LEU432 (Figure 11C).

In summary, ε-viniferin binds to critical amino acids within the APP active site in a manner comparable to FTO504 and more effectively than δ-viniferin. These molecular dynamics simulation results are consistent with docking predictions and support the ability of ε-viniferin and δ-viniferin to bind stably and specifically to the APP active site.

## 4. Discussion

Viniferin, a derivative of resveratrol and a major stilbenoid produced by plants in response to environmental stress, exists in several structural forms. Similar to other stilbene derivatives, viniferin may occur in both cis and trans configurations, although the trans form is generally considered the more stable and biologically active isomer [21]. These derivatives exhibit diverse biological activities, ranging from anti-inflammatory, antioxidant, and anticancer effects to roles in metabolic, infectious, and neurodegenerative conditions [23]. Their broad pharmacological potential highlights viniferin as a promising candidate for clinical research and future drug development, with applications spanning liver protection, cancer therapy, and chronic disease management. Chemoinformatics and drug-likeness evaluation revealed that among viniferin derivatives, ε- and δ-viniferin possess the most favorable physicochemical and pharmacological profiles (Table 1). In contrast, α-viniferin, R-viniferin, and 2-R-viniferin exhibited excessive molecular weights, lipophilicity, and hydrogen bonding capacity, failing multiple drug-like filters and thus lacking potential as viable drug-like molecules. Drug-likeness serves as an early screening tool in drug discovery, predicting whether a molecule resembles a successful oral drug rather than directly measuring efficacy. In this study, three key filters were applied: Lipinski’s Rule of Five (molecular size and solubility), Veber’s Rules (flexibility and surface area), and Egan’s Rule (absorption boundary based on LogP and PSA), to assess bioavailability potential [43]. ε- and δ-Viniferin met key drug-likeness criteria, possessing suitable molecular weights and moderate lipophilicity, which positions them as promising candidates for development. In silico pharmacokinetic and toxicological analyses highlight ε- and δ-viniferin as promising anticancer stilbenes, despite challenges related to poor solubility and low permeability. ε-Viniferin demonstrated superior predicted intestinal absorption, while both isomers exhibited low distribution volumes and limited brain penetration, thereby minimizing central nervous system (CNS) toxicity risks. Metabolic profiling revealed CYP3A4 substrate activity and CYP2C9 inhibition, with δ-viniferin also inhibiting CYP2C19. Toxicity predictions indicated no mutagenicity, hepatotoxicity, or skin sensitization, although inhibition of hERG II channels was predicted. Overall, these isomers exhibit acceptable drug-likeness and safety, warranting further validation, whereas other viniferin forms appear less viable. Pharmacokinetic data of viniferin in rats, especially ε- and δ-viniferin, indicate very low oral bioavailability (<3%) due to poor absorption and extensive first-pass metabolism. ε-Viniferin accumulates in adipose tissue and is mainly excreted via bile [44]. Encapsulation in multilamellar liposomes (MLL) has been reported to improve pharmacokinetics in rats by extending the half-life of its glucuronide metabolite, increasing tissue exposure, and enhancing stability in active organs [45]. Human safety data on viniferin are currently limited to topical use, where it shows excellent skin tolerance and no cytotoxicity at clinical doses [18]. Its systemic safety in humans remains unknown, posing a key gap in translational research. Given strong preclinical evidence, particularly in liver and cancer models, thorough toxicological and pharmacokinetic studies are required to advance viniferin derivatives toward therapeutic development [44].

Liver cancer represents a major global health burden, with over 866,000 new cases in 2022, ranking sixth worldwide and serving as the leading cause of cancer deaths in Thailand. Key risk factors include chronic HBV/HCV infection, alcohol use, and metabolic dysfunction-related liver disease. The disease typically develops from liver damage and inflammation, progressing to fibrosis and cirrhosis, which eventually drive HCC [46]. This study investigates viniferin, a natural stilbene with diverse biological activities, including anti-inflammatory, anti-angiogenic, anti-obesity, and anticancer effects [44]. Viniferin shows potential against multiple cancers by inducing apoptosis, cell cycle arrest, ROS production, and synergizing with chemotherapy, with 2R-viniferin acting via a p53-dependent mechanism [24]. Using network pharmacology, molecular docking, and MD simulations, this study sought to clarify viniferin’s mechanisms in MS, FLD, CH, and HCC. Network Pharmacology has been widely applied as a predictive analytical approach for complex diseases such as cancer [47]. We employed a framework including target identification, GO/KEGG functional analysis, and PPI analysis. After constructing a PPI network of 247 identified overlapping targets, GO analysis revealed that viniferin is associated with protein kinase activity and protein serine/threonine/tyrosine kinase activity (Figure 4). Viniferin appears to function in a multi-target manner, which is advantageous for managing the complexity of cancer cells [48]. KEGG pathway analysis indicated that viniferin is involved in cancer-related pathways and the PI3K-Akt signaling axis, both of which are critical in liver cancer development (Figure 5). Additionally, α-viniferin, a resveratrol trimer, has been noted to inhibit cancer-related pathways by targeting the TGF-β receptor, VEGFR-2, and FPR1, resulting in anti-angiogenic and anti-inflammatory effects [27]. Viniferin is a natural compound with strong potential to be developed as a targeted therapy for liver cancer in the future.

Using the cytoHubba plug-in, ten hub proteins were identified as significant: *AKT1*, *HSP90AA1*, *ESR1*, *HIF1A*, *NFKB1*, *GSK3B*, *PTGS2*, *APP*, *MTOR*, and *PIK3CA* (Figure 3B). In HCC, viniferin was found to act on multiple critical proteins involved in disease initiation. Notably, AKT1 enhances cancer cell survival by suppressing PTEN and activating Notch1, while also regulating Bcl-2 and Cyclin D1 to inhibit apoptosis. Inhibition of AKT1 reduces Notch1, Bcl-2, and Cyclin D1 expression, thereby inducing cell death and limiting tumor growth, highlighting AKT1 as a key therapeutic target [49]. Heat Shock Protein 90 Alpha 1 (HSP90AA1) stabilizes multiple oncogenic proteins, enabling cancer cell growth, apoptosis evasion, and angiogenesis. It is also linked to ER stress, metastasis, and resistance to Lenvatinib, and is regulated by ac4C mRNA modification via NAT10 [50]. Estrogen Receptor-α (ESR1) plays a protective role against liver cancer, reducing tumor risk and burden in female mice. Its absence shifts hepatic gene expression toward a male pattern, while its induction of lncRNA MEG3 suppresses proliferation and metastasis [51]. Hypoxia-Inducible Factor 1 Alpha (HIF1A) promotes HCC progression, with elevated expression linked to poor prognosis. It enhances invasion via IL-8/NF-κB, RIT1, and AKT pathways, induces angiogenesis, and supports glycolysis via the HIF-1α/PPAR-γ/PKM2 axis [52]. NF-κB subunit 1 (NFKB1) plays a major role by activating anti-apoptotic genes in hepatocytes and promoting chronic inflammation via cytokine release from Kupffer cells, leading to a tumor-promoting microenvironment [53]. Glycogen Synthase Kinase 3 beta (GSK3B) is involved in multiple signaling pathways (IGF, Notch, Wnt/β-catenin) affecting transformation. A key mechanism is the PI3K/AKT pathway, which inactivates GSK-3β, leading to β-catenin and Snail accumulation [54]. Prostaglandin-endoperoxide synthase 2 (PTGS2/COX-2) promotes HCC growth, with elevated expression observed in Hep3B and SNU-387 cells. Treatment with NS-398 or COX-2 knockdown via siRNA has been shown to reduce prostaglandin E2 (PGE_2_) levels, inhibit proliferation, and trigger apoptosis [55]. Amyloid Precursor Protein (APP) is highly expressed in liver cancer and promotes proliferation, migration, and invasion via intracellular signaling. APP also protects cancer cells from 5-FU-induced apoptosis and activates MAPK signaling [56]. APP expression is regulated by HDAC1, and HDAC1 knockdown reduces APP levels and induces apoptosis via caspase-7 [56]. MTOR plays a central role through mTORC1 and mTORC2 complexes, promoting synthesis and inhibiting autophagy. Overexpression of the mTOR pathway correlates with poor prognosis [57]. PIK3CA, when mutated or overexpressed, activates the PI3K/AKT/mTOR cascade, leading to metabolic reprogramming to support tumor growth [58]. Although viniferin demonstrates statistically significant associations with all ten identified hub proteins, the exact molecular mechanisms underlying its interactions remain insufficiently understood. Specifically, it is unclear how viniferin binds to these targets at the structural level, modulates their activity, and subsequently influences downstream signaling pathways. Further experimental validation and mechanistic studies are therefore required to elucidate how viniferin exerts inhibitory effects on these proteins and contributes to its potential anticancer activity.

Analysis using molecular docking revealed that ε- and δ-viniferin effectively inhibited APP, AKT1, and HSP90AA1 (Table 3). APP was inhibited more strongly by both compounds than by the reference drug. ε-Viniferin formed multiple hydrogen bonds with residues LYS76, TYR77, HIS81, LYS380, LYS430, and ALA434, as confirmed by 200 ns MD simulations (Figure 6 and Figure 9). δ-Viniferin showed stable interactions with GLN276 and ALA431 (Figure 10). These findings indicate that viniferin engages multiple residues through hydrogen bonding, supporting its stable binding against APP. Previous work suggests APP knockdown suppresses growth and increases sensitivity to TRAIL and 5-FU [59]. Furthermore, APP overexpression upregulates mesenchymal markers like vimentin, suggesting that APP contributes to invasiveness via MAPK signaling [60]. ε-Viniferin has also been shown to inhibit EMT in lung cancer by downregulating Snail and Zeb1 while suppressing MMP-2/9 signaling [61]. In 5-FU-resistant HCC lines, APP overexpression blocks the mitochondrial apoptosis pathway, contributing to chemoresistance [62]. The functional association between APP and HDAC1 regulation in liver cancer further supports APP as a potential therapeutic target [63].

Regarding AKT1 and HSP90AA1, previous studies have shown that ε-viniferin acts as a reversible antagonist of formyl peptide receptor 1, suppressing AKT phosphorylation [64]. α-Viniferin suppresses inflammation by inhibiting AKT/PI3K-dependent NF-κB activation [65]. Viniferin also exhibits hepatoprotective effects by modulating oxidative stress via the Nrf2 pathway and suppressing pro-inflammatory cytokines like TNF-α and IL-6 [46]. AKT activation supports cell cycle progression [66], but ROS can disrupt AKT-HSP90 binding, inducing apoptosis [67]. Both α- and ε-viniferin promote apoptosis in cancer cells by enhancing cleaved caspase-3 [68]. ε-Viniferin promotes hepatocyte survival by upregulating Bcl-2 in certain contexts [69], but when combined with vincristine, it induces apoptosis in HepG2 cells [24]. Additionally, α-viniferin was shown to inhibit K562 cell growth through modulation of caspases and HSP90AA1 [22].

**Figure 12:**
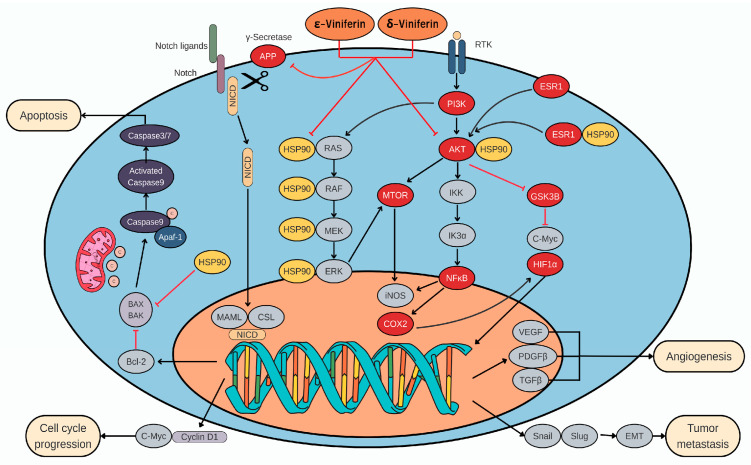
Summary of the mechanisms of viniferin based on network pharmacology, molecular docking, and MD simulation analyses.

Overall, this study provides an integrated in silico framework linking viniferin’s physicochemical properties and multi-target interactions with pathways relevant to liver cancer and metabolic disorders. Figure 12 presents a schematic overview of these proposed molecular interactions, highlighting the involvement of APP, AKT1, and HSP90AA1. While the findings support the potential of ε-viniferin and δ-viniferin, they should be interpreted as hypothesis-generating given the limitations of low oral bioavailability and the absence of direct experimental validation. Future in vitro and in vivo models will be essential to confirm target-specific mechanisms and evaluate translational feasibility.

## 5. Conclusions

This study indicates that ε- and δ-viniferin satisfy key drug-likeness criteria, including compliance with Lipinski’s Rule of Five, and exhibits generally favorable predicted pharmacokinetic and safety profiles. In silico analyses suggested good gastrointestinal absorption, limited blood–brain barrier penetration, and no predicted carcinogenicity, hepatotoxicity, or allergenicity, although potential cardiovascular concerns, such as hERG II inhibition, should be further evaluated. GeneCards-based analysis identified 247 overlapping targets shared among metabolic syndrome, fatty liver disease, chronic hepatitis, and liver cancer, supporting the relevance of viniferin to liver-associated disorders. Network pharmacology highlighted ten hub proteins, including *AKT1*, *HSP90AA1*, *ESR1*, *HIF1A*, *NFKB1*, *GSK3B*, *PTGS2*, *APP*, *MTOR*, and *PIK3CA*, that are involved in cancer-related signaling pathways. Molecular docking and molecular dynamics simulations demonstrated that viniferin interacts most consistently with APP, AKT1, and HSP90AA1, with APP showing the most stable binding behavior. Collectively, these findings suggest that viniferin may act through multi-target modulation of pathways relevant to liver disease and HCC and provide a computational basis for future experimental validation.

## Figures and Tables

**Table 1:** Analysis of chemical properties and drug-likeness of viniferin performed with SWISS ADME tools.

Molecule Properties	Compound				
	ε-Viniferin	α-Viniferin	δ-Viniferin	R-Viniferin	2-R-Viniferin
PubChem CID	5315232	196402	637098	165359521	46230190
Molecular Weight (<500 g/mol)	454.478	678.693	454.478	906.94	906.94
LogP (<5)	5.6506	8.0304	5.6506	11.0042	10.7216
No. of Rotatable Bonds	4	3	4	8	7
No. of H bond acceptors (<10)	6	9	6	12	12
No. of H bond donors (<5)	5	6	5	9	10
Surface Area (TPSA < 140 Å)	195.452	290.989	195.452	388.532	388.213
Drug Likeness (3/5)	Yes	No	Yes	No	No
Lipinski	Yes	No	Yes	No	No
Ghose	No	No	No	No	No
Veber	Yes	No	Yes	No	No
Egan	Yes	No	Yes	No	No
Muegge	No	No	No	No	No

**Table 2:** Pharmacokinetic property analysis of epsilon (ε)-viniferin and delta (δ)-viniferin using the PkCSM program.

Property	Model Name	Predicted Value	
		ε-Viniferin	δ-Viniferin
Absorption	Water solubility (log mol/L)	−2.954	−2.962
	Caco2 permeability (log Papp in 10^−6^ cm/s)	−0.511	−0.989
	Intestinal absorption (human) (% Absorbed)	96.063	87.907
	Skin Permeability (log Kp)	−2.735	−2.735
	P-glycoprotein substrate (Yes/No)	Yes	Yes
	P-glycoprotein I inhibitor (Yes/No)	Yes	Yes
	P-glycoprotein II inhibitor (Yes/No)	Yes	Yes
Distribution	VDss (human) (log L/kg)	−2.023	−2.013
	Fraction unbound (human) (Fu)	0.126	0.109
	BBB permeability (log BB)	−0.854	−1.188
	CNS permeability (log PS)	−2.823	−2.837
Metabolism	CYP2D6 substrate (Yes/No)	No	No
	CYP3A4 substrate (Yes/No)	Yes	Yes
	CYP1A2 inhibitor (Yes/No)	No	No
	CYP2C19 inhibitor (Yes/No)	No	Yes
	CYP2C9 inhibitor (Yes/No)	Yes	Yes
	CYP2D6 inhibitor (Yes/No)	No	No
	CYP3A4 inhibitor (Yes/No)	No	No
Excretion	Total Clearance (log mL/min/kg)	−0.026	−0.101
	Renal OCT2 substrate (Yes/No)	No	No
Toxicity	AMES toxicity (Yes/No)	No	No
	Max. tolerated dose (human) (log mg/kg/day)	0.409	0.408
	hERG I inhibitor (Yes/No)	No	No
	hERG II inhibitor (Yes/No)	Yes	Yes
	Oral Rat Acute Toxicity (LD_50_) (mol/kg)	2.568	2.606
	Oral Rat Chronic Toxicity (LOAEL) (log mg/kg bw/day)	3.267	3.309
	Hepatotoxicity (Yes/No)	No	No
	Skin Sensitization (Yes/No)	No	No
	*T. pyriformis* toxicity (log μg/L)	0.285	0.285
	Minnow toxicity (log mM)	1.161	0.503

**Table 3:** Binding energy values and inhibition constants of epsilon (ε)-viniferin and delta (δ)-viniferin from in silico molecular docking, implicated in liver cancer pathogenesis.

Protein Target	PDB ID	Compounds	Binding Energies (kcal/mol)	Inhibition Constant
AKT1	8UW7	ε-Viniferin	−10.44	22.22 nM
		δ-Viniferin	−10.86	10.91 nM
		XOO502	−12.24	1.08 nM
HASP90AA1	5CF0	ε-Viniferin	−10.39	24.05 nM
		δ-Viniferin	−11.41	4.33 nM
		FJS301	−11.54	3.49 nM
ESR1	6VPF	ε-Viniferin	−5.86	50.23 µM
		δ-Viniferin	−6.33	23.03 µM
		Clomifene	−5.05	197.47 µM
HIF1A	1H2M	ε-Viniferin	−6.05	36.8 µM
		δ-Viniferin	−7.14	5.85 µM
		BAY 87-2243	−7.58	2.78 µM
NFKB1	8TQD	ε-Viniferin	−5.66	70.7 µM
		δ-Viniferin	−6.03	37.84 µM
		JMR301	−4.30	704.68 µM
GSK3B	5F94	ε-Viniferin	−9.85	60.44 nM
		δ-Viniferin	−9.76	70.68 nM
		3UO401	−7.07	6.58 nM
PTGS2	5IKR	ε-Viniferin	−8.55	543 nM
		δ-Viniferin	−9.76	69.54 nM
		mefenamic acid	−7.57	2.81 µM
APP	7D8X	ε-Viniferin	−10.01	46.34 nM
		δ-Viniferin	−11.12	7.11 nM
		FTO504	−8.14	1.09 µM
MTOR	5GPG	ε-Viniferin	−9.17	190.96 nM
		δ-Viniferin	−9.25	165.67 nM
		RAP301	−10.50	20.12 nM
PIK3CA	5DXT	ε-Viniferin	−8.84	330.03 nM
		δ-Viniferin	−9.73	74.37 nM
		5H51101	−10.13	37.62 nM

## Data Availability

The original contributions presented in this study are included in the article. Further inquiries can be directed to the corresponding author.

## Abbreviations

The following abbreviations are used in this manuscript:

Å	Angström
AKT1	Protein kinase B alpha
AMES	Ames mutagenicity test
APP	Amyloid precursor protein
ASR	Age-standardized rate
BBB	Blood–brain barrier
BP	Biological process
CADD	Computer-aided drug design
CC	Cellular component
CH	Chronic hepatitis
COX-2	Cyclooxygenase-2
CYP	Cytochrome P450
DAMPs	Damage-associated molecular patterns
EMT	Epithelial–mesenchymal transition
FLD	Fatty liver disease
FDR	False discovery rate
GO	Gene Ontology
GOBP	Gene Ontology Biological Process
GOCC	Gene Ontology Cellular Component
GOMF	Gene Ontology Molecular Function
GPx	Glutathione peroxidase
HBV	Hepatitis B virus
HCV	Hepatitis C virus
HCC	Hepatocellular carcinoma
HDAC	Histone deacetylase
HIF1A	Hypoxia-inducible factor 1 alpha
HSP90AA1	Heat shock protein 90 alpha family class A member 1
IGF	Insulin-like growth factor
KEGG	Kyoto Encyclopedia of Genes and Genomes
MAPK	Mitogen-activated protein kinase
MASLD	Metabolic dysfunction-associated steatotic liver disease
MD	Molecular dynamics
MF	Molecular function
MLL	Multilamellar liposomes
MS	Metabolic syndrome
MTOR	Mechanistic target of rapamycin
NFKB1	Nuclear factor kappa B subunit 1
OCT2	Organic cation transporter 2
PAMPs	Pathogen-associated molecular patterns
PDB	Protein Data Bank
PI3K	Phosphoinositide 3-kinase
PIK3CA	Phosphatidylinositol-4,5-bisphosphate 3-kinase catalytic subunit alpha
PPI	Protein–protein interaction
PSA	Polar surface area
RMSD	Root mean square deviation
RMSF	Root mean square fluctuation
RO5	Rule of Five
ROS	Reactive oxygen species
SEA	Similarity ensemble approach
SMILES	Simplified molecular-input line-entry system
SOD	Superoxide dismutase
TPSA	Topological polar surface area
VDss	Volume of distribution at steady state
